# Ablation index-guided ablation with milder targets for atrial fibrillation: Comparison between high power and low power ablation

**DOI:** 10.3389/fcvm.2022.949918

**Published:** 2022-08-04

**Authors:** Zheng Liu, Li-feng Liu, Xiao-qin Liu, Jiapeng Liu, Yu-xin Wang, Ye Liu, Xing-peng Liu, Xin-chun Yang, Mu-lei Chen

**Affiliations:** Heart Center, Beijing Chaoyang Hospital, Capital Medical University, Beijing, China

**Keywords:** atrial fibrillation, high power, ablation index, pulmonary vein isolation, catheter ablation

## Abstract

**Background:**

High power-ablation index (HP-AI)-guided ablation for atrial fibrillation (AF) targeting high AIs has been implemented in European countries. However, milder AI targets are widely used in Asia. The safety and efficacy of HP-AI-guided ablation compared with those of low-power AI-guided ablation in a milder AI-targeting setting are unknown. The goal of this study was to explore the efficacy and safety of HP-AI-guided ablation in a milder AI-targeting setting.

**Methods:**

Patients who underwent pulmonary vein isolation (PVI) for AI-guided atrial fibrillation ablation in our center were enrolled and divided into 2 groups according to the ablation power used. In the HP-AI group, the ablation power was over 45 W, while the low power-AI group was ablated with <35 W power. The targeted AIs were 450–500 in the anterior wall and 350–400 in the posterior wall. The efficacy outcome was expressed as the single-procedure atrial arrhythmia-free survival between 91 days and 1 year. Safety outcomes included severe adverse events (SAEs), including symptomatic pulmonary vein (PV) stenosis, atrioesophagal fistula, cardiac tamponade, stroke, thromboembolism events, myocardial infarction, and major bleeding.

**Results:**

A total of 134 patients were enrolled, of whom 74 underwent PVI using HP-AI, while 60 received low power-AI ablation. After a mean follow-up time of 7.4 months, 22 (16.4%) patients showed arrhythmia recurrence: 5 (6.8%) patients in the HP-AI group and 17 (28.3%) patients in the low power-AI group. The HP-AI group showed a significantly higher arrhythmia-free survival than the low power-AI group (*p* = 0.011). Two patients in the low power-AI group and 1 patient in the HP-AI group developed an SAE (*p* = NS). Compared with the low power-AI group, the HP-AI group demonstrated a higher PV first-pass isolation rate, shorter ablation time, and fewer patients with anatomical leakages and sites of unreached AI.

**Conclusion:**

In a milder AI setting, HP-AI ablation might result in significantly higher arrhythmia-free survival than low power-AI ablation and a similar safety profile.

## Introduction

Atrial fibrillation (AF) ablation is an effective method for controlling symptoms in AF patients. While it has evolved considerably in the past two decades, pulmonary vein isolation (PVI) has been established as the cornerstone for the invasive treatment of AF (1). Late reconnection of the pulmonary veins (PV) after ablation leads to recurrence of AF (2). Good ablation lesion quality is crucial for achieving durable isolation of the PV. With the utility of a new lesion surrogate, the ablation index (AI), calculated by integrating ablation time, energy, catheter stability, and contact force, several studies have identified that AI-guided ablation is superior to contact force-guided (3, 4) or other forms of lesion surrogate-guided ablation (5). High AI ablation targeting an AI of 550 for the PV anterior wall and 400 for the PV posterior wall with an inter-lesion distance (ILD) <6 mm, also called the “CLOSE” protocol, has been used as a reference for many other studies (4, 6, 7). Most ablations under the CLOSE protocol are performed using low power, ranging between 30 and 35 W (3, 4). For more efficient ablation, high-power (HP) ablation with radiofrequency application between 45 and 55 W have also evolved for AF ablation and has been found to be safe (8–10) and efficient (11, 12) in European AF patients. However, a narrower safety margin has also been observed when using high-power ablation targeting high AIs with the CLOSE protocol (6). In addition, milder AI targeting and conventional ablation power applications are more commonly used in Asia (13–15). In this study, we attempted to evaluate the safety and efficacy of HP-AI-guided ablation in a milder AI-targeted setting compared with low-power AI-guided ablation.

## Materials and methods

### Study design and study population

In this prospective study, all patients who underwent catheter ablation for AF were admitted to the hospital from December 2020 to December 2021. The enrollment criteria were as follows: (1) Eligible patients aged 18 to 80 years with documented symptomatic non-valvular AF episodes receiving their first radiofrequency catheter ablation guided by AI. (2) Patients with paroxysmal AF and very short period persistent AF patients (with evidence of AF last <1 month), in whom only PVI was performed in the left atrium and the need for no other substrate modifications. The exclusion criteria were as follows: (1) Uncontrollable hypertension (systolic blood pressure (SBP) > 180 mmHg or diastolic blood pressure (DBP) > 110 mmHg with antihypertensive medications). (2) New York Heart Association function class IV, left ventricular ejection fraction <30%. (3) Terminal illnesses with life expectancy <1 year. (4) Prior AF catheter ablation. (5) Contraindications to anticoagulation.

The study population was divided into two groups based on the radiofrequency ablation power used, i.e., the high-power group (≥45 W, HP-AI) and the low-power group (≤ 35 W, low power-AI). The subjects were grouped depends on the order of the operation. The first operation on each operation day is high-power ablation, followed by low-power ablation, and back and forth in this order. And all the study subjects were enrolled consecutively.

The collected data included demographic characteristics, procedure-related characteristics, and clinical outcomes. All patients signed informed consent. The study protocol conforms to the ethical guidelines of the 1975 Declaration of Helsinki as reflected in a prior approval by the human research committee of the institution.

### Preprocedure preparation

Transesophageal echocardiography was performed before the procedure to rule out intracardiac thrombus. For patients who underwent vitamin K antagonist (VKA) therapy, the VKA was uninterrupted, targeting an international normalized ratio (INR) of 2–2.5. For patients who underwent non-vitamin K antagonist oral anticoagulant (NOAC) therapy, the morning dose was skipped and resumed 6 h after the procedure.

### Mapping and ablation procedure

All PVI procedures were performed by one experienced operator, who had individually performed more than 1,000 AF ablation cases in the last 5 years, consistently using the standardized institutional approach. The procedure was performed under conscious sedation using fentanyl and midazolam. A surface electrocardiogram (ECG) and bipolar endocardial electrograms were continuously monitored and recorded with a computer-based digital amplifier and recording system (Bard Electrophysiology). After placing a 6F decapolar catheter into the coronary sinus and an 11F intracardiac echocardiography (ICE) catheter into the right atrium, transseptal puncture was performed under ICE guidance using a modified Brockenbrough technique. One 8.5F sheath (SL1, Abbott, USA) was introduced into the left atrium. After transseptal puncture, 100 units/kg heparin was injected into the left atrium (LA), followed by repeated injections of heparin every 15–20 min to maintain an activated clotting time of 250–300 s during the procedure.

Three-dimensional reconstruction of the LA geometry was performed using an electroanatomical mapping system (CARTO 3; Biosense Webster Inc.) with a steerable five-spine 20-pole mapping catheter (PentaRay, Biosense Webster, USA; interelectrode spacing 2-6-2 mm) and a 3.5-mm open-irrigated tip contact force-sensing ablation catheter (Thermocool SmartTouch or SmartTouch surround flow; Biosense-Webster Inc.). The ostium of the ipsilateral pulmonary vein was tagged in the LA geometry for ablation guidance. The PVI ablation lesion was created at the ostium of the pulmonary vein in a point-by-point fashion using the ablation catheter. For high-power ablation (≥45 W), a surround flow-irrigated catheter was used. For lower power ablation (all ablated with 35 W), a 6-hole irrigated catheter or surround flow-irrigated catheter was used at the operator's discretion. The power setting was not changed during an individual PVI procedure. All ablations were performed to target an AI of 450–500 for the anterior segments and 350–400 for the posterior wall. All RF applications were depicted on the electroanatomic map with automated tags (CARTO VISITAG, Biosense Webster Inc., Diamond Bar, CA, USA). The Visitag settings were as follows: catheter position stability: minimum force 5 g, minimum time 5 s, force-overtime 30%, maximum range 4 mm, and lesion-tag size 2 mm. First-pass isolation (FPI) was defined as isolation of ipsilateral PV that occurred either before or at the completion of the circumferential lesion set, without the need for ablation of the PV carina or additional segmental ablation. If reconnection was found, reisolation was performed by targeting the earliest activated PV potential. Following confirmation of PVI, bipolar pacing (output 10 mA, pulse width 2 ms) was performed with the ablation catheter just inside the ablation line to identify excitable gaps. Acute PVI success was defined as achievement of entrance block into all PV under an isoproterenol/ATP challenge 30 min after PVI. In all enrolled patients, ablation was restricted to PVI, regardless of AF type, except for patients with documented typical right atrial flutter for whom additional cavotricuspid isthmus (CTI) ablation was performed.

### Ablation and procedure data

The total ablation time was counted offline as the accumulation of the ablation times of all lesion tags. For all patients, the number of lesions created to achieve PVI, the mean ablation time, contact force, and AI of each lesion were documented for further comparison. During the PVI procedure, the ILD was not standardized and was determined at the operator's discretion. However, an ILD over 5 mm was noted and defined as the presence of anatomic leakage. In addition, lesions with an AI below the target were also noted.

### Postablation care and follow-up

Patients were monitored continuously with transtelephonic electrocardiography for 24 h in the hospital. Pericardial effusion was ruled out by transthoracic echocardiography. Patients was given a proton pump inhibitor (PPI) and anticoagulant for 2 months after the procedure. Antiarrhythmic drugs (AADs) were prescribed if atrial arrhythmia was documented *via* in-hospital ECG monitoring and were stopped 1 month after the procedure. If chest discomfort was reported, contrast-enhanced X-ray computed tomography was performed to detect PV stenosis and/or atrioesophageal fistula. Cerebral magnetic resonance imaging was performed for any patients who had newly developed neurological symptoms to identify possible stroke.

A blanking period of 3 months was allowed. The follow-up data were obtained at 3, 6, and 12 months after the procedure.

### Study outcome

The primary efficacy outcome was expressed as the single-procedure atrial arrhythmia-free survival between 91 days and 1 year, which was defined as freedom from any AF or atrial tachycardia (AT) episodes >30 s as recorded by electrocardiogram, transtelephonic electrocardiographic monitoring, 24 h Holter monitoring, or telemetry. Short-term procedure-related efficacy outcomes included the acute PVI success rate and FPI rate. Safety outcomes included severe adverse events (SAEs), including symptomatic PV stenosis, atrioesophageal fistula, cardiac tamponade, stroke, thromboembolism events, myocardial infarction, and major bleeding.

### Statistical analysis

Continuous variables are reported as the mean ± SD or the median and 25^th^ and 75^th^ percentiles according to their distribution. Categorical data are expressed in terms of frequency and percentage. The Kolmogorov–Smirnov test was performed to assess the normality of continuous variables. Student's *t* test was performed to assess the differences in continuous, normally distributed, and homoscedastic data between the two groups; the Mann–Whitney test was used otherwise. Fisher's chi-square test was performed to investigate the relationships between dichotomous variables. Pearson's chi-square test was performed to investigate the relationships between grouping variables. Kaplan–Meier analysis was used to assess freedom from AF/AT during the follow-up. Cox regression was used to assess the influence on the arrhythmia-free survival of the procedures. For all tests, a *P*-value < 0.05 (two tailed) was considered significant. All statistical analyses were performed using SPSS software (version 23.0, SPSS Inc.).

## Results

### Patient demographic characteristics

The demographic information and baseline data of the 134 study participants are provided in Table 1.

**Table 1:** Characteristics of the HP-AI group and the low power-AI group.

	**All PVI patients** ***n*** = **134**	**HP-AI PVI** ***n*** = **74**	**Low power-AI PVI** ***n*** = **60**	***P*** **-value**
Age, years	66.6 ± 9.7	66.7 ± 11.3	66.4 ± 7.2	0.846
Female, *n* (%)	51 (38.1)	28 (37.8)	23 (38.3)	0.953
BMI, kg/m^2^	23.35 ± 2.51	23.16 ± 2.63	23.59 ± 2.31	0.322
Paroxysmal AF, *n* (%)	108 (80.6)	57 (77.0)	51 (85.0)	0.246
LA diameter, mm	39.5 ± 4.6	40.8 ± 3.8	38.1 ± 5.0	0.027
CHA2DS2-VASC score	2.9 ± 1.8	2.7 ± 1.9	3.1 ± 1.8	0.264
Diabetes, *n* (%)	43 (32.1)	20 (27.0)	23 (38.3)	0.194
Hypertension, *n* (%)	71 (53.0)	43 (58.1)	28 (46.7)	0.224
Coronary heart disease, *n* (%)	66 (49.3)	33 (44.6)	33 (55.0)	0.153
Stroke, *n* (%)	12 (9.0)	7 (9.5)	5 (8.3)	1.000
Sinus bradycardia, *n* (%)	10 (7.5)	6 (8.1)	4 (6.7)	1.000
CrCl <80 ml/min, *n* (%)	12 (9.0)	6 (8.1)	6 (10.0)	0.703
Failed AADs, *n*	1.1 ± 0.8	1.1 ± 0.8	1.1 ± 0.7	0.582
On anticoagulation, *n* (%)	103 (76.9)	60 (81.1)	43 (71.7)	0.199

PVI, pulmonary vein isolation; HP, high power; AI, ablation index; BMI, body mass index; AF, atrial fibrillation; LA, left atrium; CrCl, creatinine clearance rate; AADs, anti-arrhythmic drugs.

The mean age of the enrolled patients was 66.6 ± 9.7 years; 83 (61.9%) patients were male. The mean left atrium diameter was 39.5 ± 4.6 mm. One hundred and eight patients (80.6%) had paroxysmal atrial fibrillation at the time of enrollment. For patients who received HP-AI-guided PVI, the left atrial diameter was slightly larger than that among patients who received low power-AI-guided PVI. Other demographic conditions, comorbidities (mainly presented as CHA2DS2-VASC scores) and medication statuses were similar between the two groups of patients.

### Procedure data

**Figure 1:**
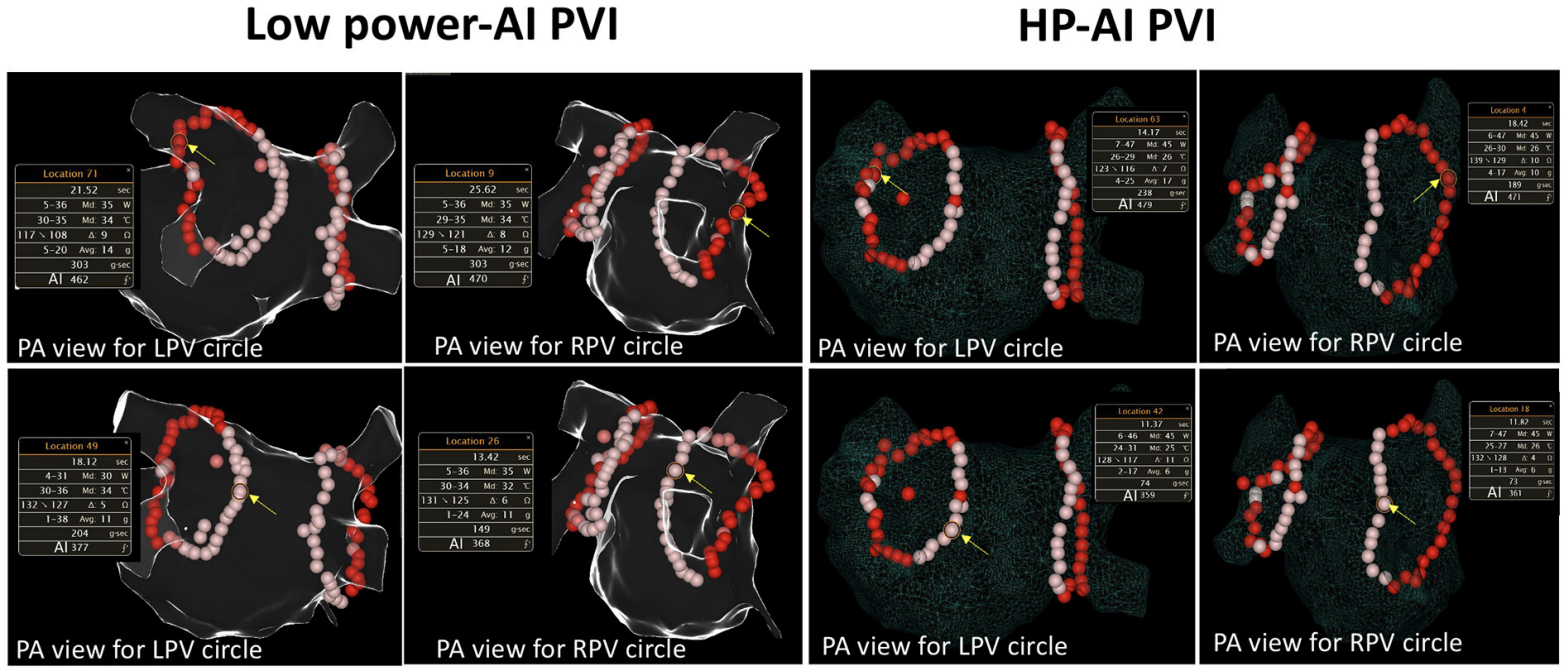
Examples of HP-AI- and low-power AI-guided PVI procedures. The detailed ablation parameters of the highlighted (with yellow arrow) lesion are presented. The red-colored-tags represent lesions with an AI ranging from 400 to 500. Tags in pink represent lesions with an AI ranging between 350 and 399. Note that the power delivered between the two groups was different (35 and 45 W, respectively). AI, Ablation index, calculated with contact force, power delivered, and ablation time. PA view, posterioanterior view; RPV, right pulmonary vein; LPV, left pulmonary vein.

Of all enrolled patients, 74 received PVI using the HP-AI technique, while 60 received low-power AI-guided ablation (Figure 1). Detailed ablation parameters of the two groups are presented in Table 2. Extrapulmonary vein trigger elimination (superior vena cava isolation) was performed more often in patients in the HP-AI group. CTI linear ablation was performed in similar proportions in the two groups. For PVI ablation, the total ablation time of the procedure and the ablation time for each lesion in the HP-AI group were much shorter than those in the low power-AI PVI group. Other parameters, including contact force for each lesion, average AI for each lesion, and lesion numbers for creating the ablation circles, were similar between the two groups.

**Table 2:** Procedure data between the HP-AI group and the low power-AI group.

	**All patients** ***n*** = **134**	**HP-AI PVI** ***n*** = **74**	**Low power-AI PVI** ***n*** = **60**	***P*** **-value**
Cavotricuspid isthmus ablation (%)	15 (11.2)	9 (12.2)	6 (10.0)	0.693
Extra PV trigger elimination (%)	12 (9.0)	10 (13.5)	2 (3.3)	0.040
Mean power used for ablation (W)	40.6 ± 5.2	45.0 ± 2.5	35.3 ± 0.6	<0.001
Total ablation time (s)	1,757 ± 497	1,504 ± 405	2,069 ± 418	<0.001
Average ablation time per lesion (s)	19.3 ± 4.9	17.1 ± 4.7	22.0 ± 3.6	<0.001
Average contact force per lesion (g)	9.2 ± 1.1	9.3 ± 1.2	9.2 ± 0.9	0.661
Average AI for LPV lesions	403 ± 29	406 ± 27	399 ± 31	0.118
Average AI for LPV anterior wall	455 ± 11	456 ± 11	454 ± 10	0.481
Average AI for LPV posterior wall	352 ± 10	353 ± 11	352 ± 9	0.383
Average AI for RPV lesions	408 ± 28	409 ± 30	408 ± 26	0.776
Average AI for RPV anterior wall	462 ± 16	463 ± 15	461 ± 17	0.527
Average AI for RPV posterior wall	357 ± 16	357 ± 13	357 ± 19	0.912
First-pass LPV lesions, *n*	41 ± 9	40 ± 9	43 ± 10	0.086
First-pass RPV lesions, *n*	43 ± 7	43 ± 7	43 ± 7	0.973
Total LPV lesions, *n*	44 ± 11	43 ± 11	45 ± 12	0.183
Total RPV lesions, *n*	48 ± 11	47 ± 10	49 ± 13	0.248
First-pass isolations for both circles, *n* (%)	65 (48.5)	46 (62.2)	19 (31.7)	<0.001
First-pass isolations for at least one circle, *n* (%)	109 (81.3)	68 (91.9)	41 (68.3)	<0.001
First-pass isolation for LPV, *n* (%)	93 (69.4)	58 (78.4)	35 (58.3)	0.012
First-pass isolation for RPV, *n* (%)	81 (60.4)	55 (74.3)	26 (43.3)	<0.001
LPV waiting period reconnection, *n* (%)	19 (14.2)	11 (14.9)	8 (13.3)	0.800
RPV waiting period reconnection, *n* (%)	15 (11.2)	8 (10.8)	7 (11.7)	0.876
Patients presenting with anatomical leakages, *n* (%)	60 (45.1)	24 (32.9)	36 (60.0)	0.002
Patients presenting with an AI below target, *n* (%)	47 (35.3)	20 (27.4)	27 (45.0)	0.035
Severe adverse events, *n* (%)	3 (2.2)	1 (1.4)	2 (3.3)	0.441

HP, high power; AI, ablation index; PVI, pulmonary vein isolation; LPV, left pulmonary veins; RPV, right pulmonary veins.

**Figure 2:**
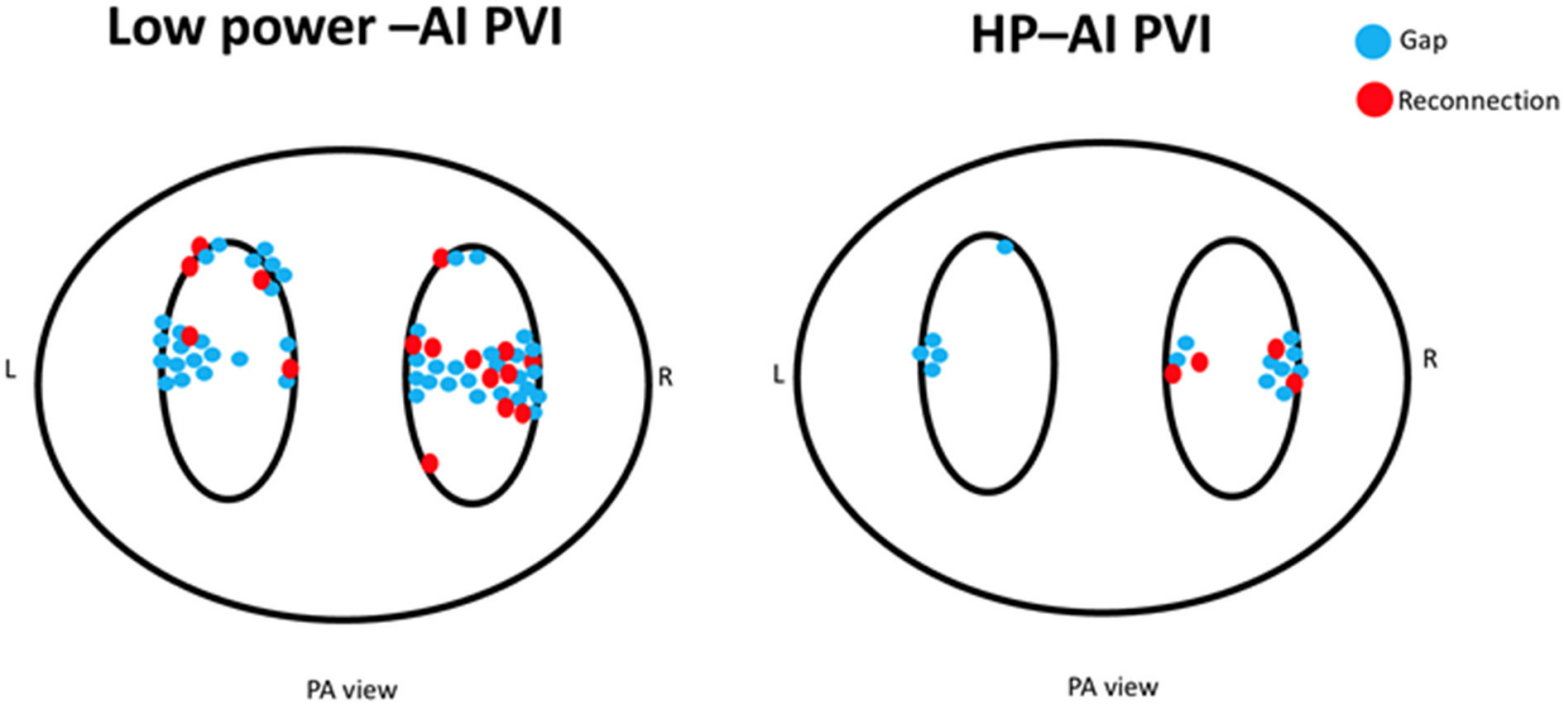
The distribution of additional lesions for closing gaps for acute PVI (blue dots) and reconnected sites for the two groups (red dots). Of note, the low power-AI group required more ablation sites to achieve PVI. The distribution of gaps and reconnections was mainly located in the carinal area between the superior and inferior veins.

The HP-AI group had a higher first-pass isolation rate for both the left and right PV circles than the low power-AI group (HP-AI vs. low-power AI: LPV first-pass isolation 78.4 vs. 58.3%, *p* = 0.012; RPV first-pass isolation 74.3 vs. 43.3%, *p* < 0.001). Acute PVI success was achieved in all patients. The distribution of additional lesions targeting residual gaps to achieve acute PVI success is presented in Figure 2. The incidence of PV reconnection was similar between groups (Table 2). The reconnected areas were mainly distributed in the carina between the superior and inferior veins for both the left and right PV (Figure 2).

**Figure 3:**
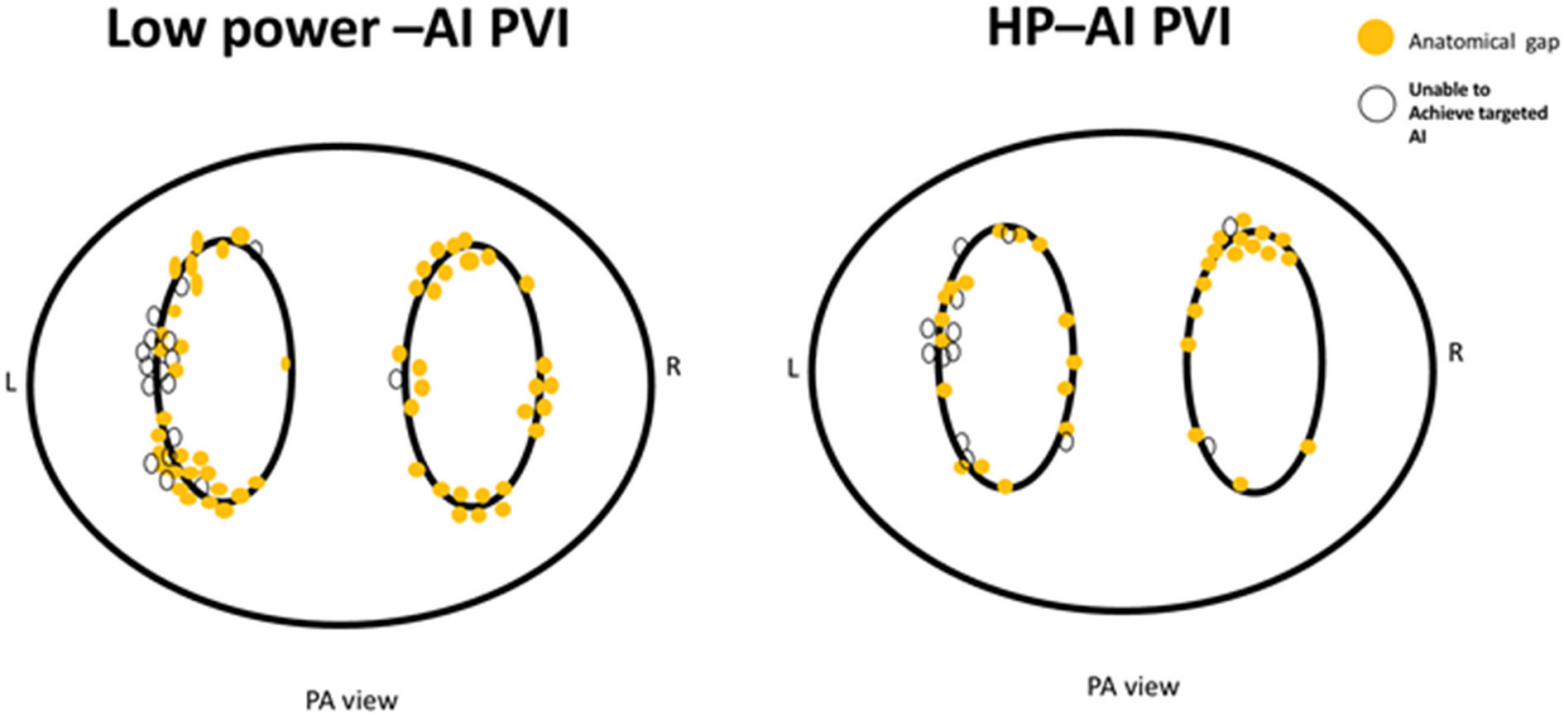
The distribution of anatomical leakages after PVI (yellow dots) and sites of below-target AI (white dots).

The AI cutoff value was not reached in 20 different patients (27.4%) in the HP-AI group and 27 different cases (45.0%) in the low power-AI group, and this difference was statistically significant. Similarly, more anatomical leakages were found in the low-power AI group (Table 2). The detailed anatomic locations of below-target AI ablation tags and anatomical leakages are presented in Figure 3.

Of note, the ridge of the left PV and roof of both the left and right PV were the main sites of clustering for anatomical leakages and below-target AI ablations, which was caused by catheter manipulation difficulties in these areas. Anatomical leakages and sites of below-target AI at the posterior wall were due to the fear of causing esophageal damage, based on the anatomical proximity or patients' complaints of more severe chest pain.

### Periprocedural and 90-day severe adverse events

Two patients in the low power-AI group developed SAEs, including 1 cardiac tamponade and 1 severe bleeding caused by femoral pseudoaneurysm. One patient in the HP-AI group developed PV stenosis because additional ablation inside the PV to eliminate the residual PV potential was implemented in this patient. No death, atrioesophageal fistula, stroke, thromboembolism event, or myocardial infarction was observed in the other patients.

### Atrial arrhythmia-free survival

During the 3-month blanking period, 31 (23.1%) patients had early recurrence, all were treated with electrical cardioversion, and clinical follow-up was performed.

**Figure 4:**
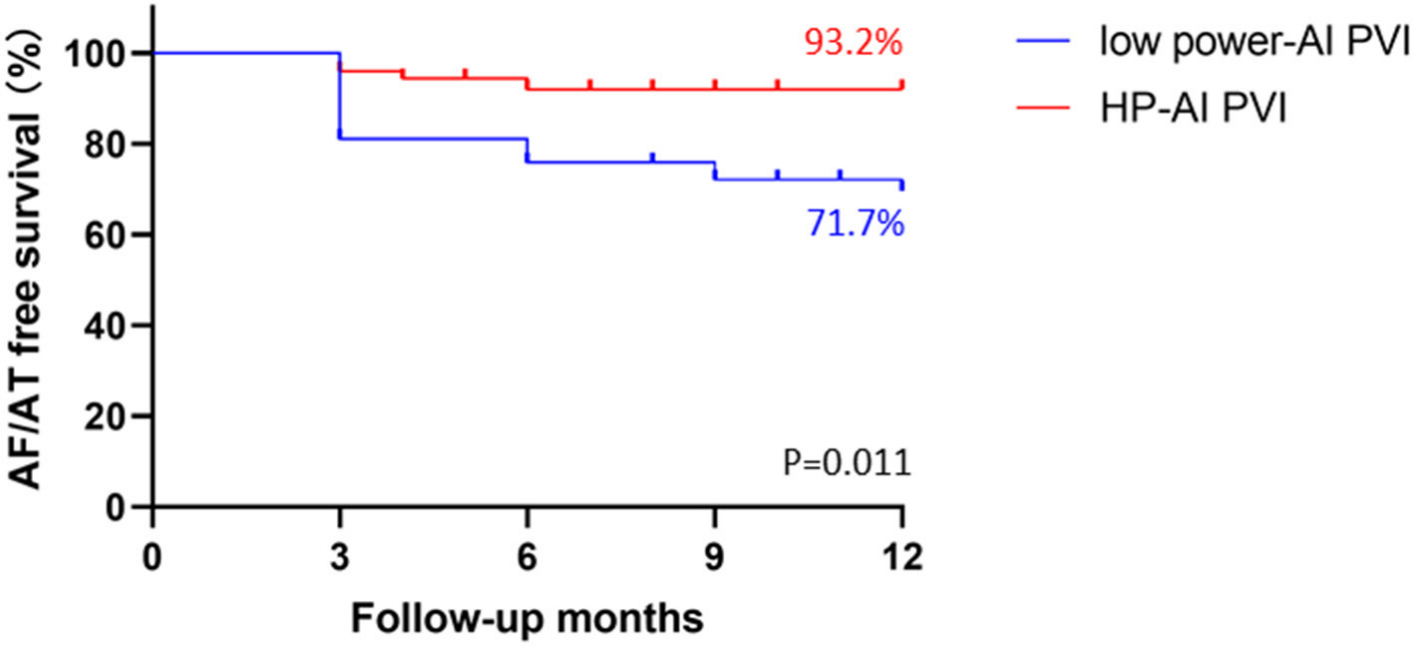
Kaplan–Meier curves of the HP-AI and low power-AI groups.

The process of clinical follow-up after the blanking period is illustrated in Figure 4. A mean follow-up time of 7.4 months was achieved in 134 patients. During the follow-up, 22 (16.4%) patients showed arrhythmia recurrence: 5 (6.8%) patients in the HP-AI group and 17 (28.3%) patients in the low power-AI group. Kaplan–Meier curves (Figure 4) showed a significantly higher arrhythmia-free survival in the HP-AI group than in the low power-AI group (*p* = 0.011).

Clinical parameters that might indicate recurrence are summarized in Table 3, and ablation parameters that might affect recurrence are summarized in Table 4. Because compared with low power-AI PVI, HP-AI PVI resulted in a shorter ablation time, higher rates of FPI of PV, and a smaller number of patients with anatomical leakages or sites of below-target AI, only the group category, other than the detailed changes resulting from different ablation power applications, was used for further analysis to determine predictors for recurrence. After multivariate analysis, the HP-AI group was associated with lower rates of recurrence (odds ratio 0.285, 95% CI 0.103–0.784, *p* = 0.015) after adjusting for CTI ablation, which seems to promote recurrence according to the present data.

**Table 3:** Clinical characteristics for recurrence.

	**AF/AT free** ***n*** = **112**	**Recurrence** ***n*** = **22**	**Hazard ratio for recurrence (95% CI)**	***P*** **-value**
Age, years	66.8 ± 9.6	65.2 ± 9.8	0.984 (0.942–1.027)	0.464
Female, *n* (%)	43 (38.4)	8 (36.4)	0.927 (0.389–2.211)	0.858
BMI, kg/m^2^	23.47 ± 2.58	22.71 ± 1.88	0.893 (0.754–1.057)	0.188
Paroxysmal AF, *n* (%)	91 (81.3)	17 (77.3)	0.668 (0.244–1.829)	0.666
LA diameter, mm	39.4 ± 4.5	40.3 ± 5.2	1.044 (0.902–1.208)	0.582
CHA2DS2-VASC	2.9 ± 1.8	2.9 ± 1.9	0.982 (0.780–1.237)	0.986
Diabetes, *n* (%)	36 (32.7)	7 (31.8)	0.954 (0.389–2.342)	0.919
Hypertension, *n* (%)	65 (58.0)	10 (45.5)	0.648 (0.280–1.500)	0.311
Coronary heart disease, *n* (%)	55 (49.1)	11 (50.0)	0.954 (0.413–2.203)	0.912
Stroke, *n* (%)	11 (9.8)	1 (4.5)	0.379 (0.051–2.831)	0.344
Sinus bradycardia, *n* (%)	7 (6.3)	3 (13.6)	2.279 (0.670–7.749)	0.187
CrCl <80 ml/min, *n* (%)	10 (8.9)	2 (9.1)	1.051 (0.245–4.504)	0.947

AF, atrial fibrillation; AT, atrial tachycardia; BMI, body mass index; LA, left atrium; CrCl, creatinine clearance rate.

**Table 4:** Ablation characteristics for recurrence.

	**AF/AT free** ***n*** = **112**	**Recurrence** ***n*** = **22**	**Univariate analysis**		**Multivariate analysis**	
			**Hazard ratio for recurrence (95% CI)**	***P*** **-value**	**Hazard ratio for recurrence (95% CI)**	***P*** **-value**
Extra PV trigger ablation, *n* (%)	11 (9.8)	1 (4.5)	0.516 (0.069–3.841)	0.518		
Cavotricuspid isthmus ablation, *n* (%)	9 (8.0)	6(27.3)	3.39 (1.321–8.715)	0.011	3.596 (1.400–9.238)	0.008
HP-AI PVI	69 (61.6)	5 (22.7)	0.297 (0.108–0.816)	0.019	0.285 (0.103–0.784)	0.015

AF, atrial fibrillation; AT, atrial tachycardia; PV, pulmonary veins; HP, high power; AI, ablation index; PVI, pulmonary vein isolation.

## Discussion

### Key findings

The major findings of the study are as follows: (1) In a milder AI-targeted setting, the HP-AI group was associated with higher rates of atrial arrhythmia freedom during an intermediate follow-up period; (2) The HP-AI group was associated with a shorter ablation time, higher rates of FPI for both the left and right PV, and a smaller proportion of patients who presented with anatomical leakages and sites with below-target AI targets; and (3) The HP-AI group had a similar rate of SAEs as the low power-AI group.

### High power in milder ablation index target guided ablation

The AI guided ablation has improved outcome considerably based on recent observations. Taghji et al. (16) used low power (25–35 W)-AI guidance and the CLOSE protocol (targeted AI of 550 for the anterior wall and 400 for the posterior wall, ILD 6 mm) for PVI and reached a FPI percentage of 98%. At 12 months, single-procedure freedom from AF/AT/AFL was 91.3% in 104 patients off antiarrhythmic drug therapy and 96.2% in 26 patients on antiarrhythmic drug therapy. Similar results were seen in the study by Phlips et al. (4), who compared AI-guided AF ablation using the CLOSE protocol targeting high AIs (550 for the anterior wall and 400 for the posterior wall) by low-power (35 W) ablation with contact force-guided ablation. Significantly higher FPI percentages (98 vs. 54%, *p* < 0.001) and one-year AF freedom survival rates (94 vs. 80%, *p* < 0.05) were observed in the AI-guided ablation group. Chen et al. (12) performed high-AI target CLOSE protocol-guided ablation using high power (over 45 w) for PVI in atrial fibrillation patients. The FPI rate was 96.7%, and the 15-month AF freedom survival rate was 85.2%. Wielandts et al. (6) randomized patients who underwent high-AI target CLOSE protocol-guided ablation into two groups who were ablated using low power (35 w) and high power (45 w). The 6-month AF recurrence rates were 8 and 10%, respectively, which were not significantly different between the groups. While the CLOSE protocol with high-AI target-guided ablation is widely used in Western countries (4, 10, 16, 17), milder AI targeting is more commonly used in Asian countries (13, 14). The possible reasons for this are as follows: (1) Asians have a smaller atrium than people of other ethnicities (18); (2) An aggressive AI might be associated with a higher risk of complications (6, 19); and (3) The threshold AI for no reconnection in the anterior wall and roof are 480 and 370 for the posterior wall and floor, respectively, according to the study by Das et al. (20). In this study, we compared high power and low power in a setting involving milder targeted AIs, which were 450–500 for the anterior wall and 350–400 for the posterior wall. We observed that FPI for at least one PV was achieved in 91.9% of patients in the HP-AI group, which was significantly higher than that in the low power-AI group (68.3% for at least one PV). Of greater importance, we observed a higher AF/AFL-free survival in the HP-AI group (93.8%) than in the low-power-AI group (71.7%) during a mean follow-up time of 7.4 months. These findings were in accordance with a previous study showing that a high FPI rate was associated with a higher probability of remaining 1-year AF free (21). The better clinical outcome could be the result of better lesion quality using high power. Recent studies found differences of lesion geometries in same AI but variate power applied (22, 23). In general, high-power lesions were wider than low-power lesions. In this study, we observed that fewer patients in the HP-AI group had anatomical leakages or sites of below-target AI. In addition, a high rate of FPI was also found in patients treated with HP-AI-guided ablation, in accordance with a previous study (15). This can be the result of different lesion formation process under different ablation power. As in previous study (23), we also observed that lesion formation was much faster in HP-AI ablation group. In a beating heart, stabilizing the catheter for longer period at a point is obviously much more difficult than ablation for much shorter time. Especially when the patient does not have mechanical ventilation under general anesthesia, the irregular breathing movement caused by pain will easily interrupt the AI calculation, making the targeted AI unreachable. This is more likely to occur in long-term ablation than in short-term ablation. In addition, not only the lesion geometries, and the ablation time, but also the biophysics of ablation might influence lesion quality. Radiofrequency ablation lesions form mostly during the initial period of ablation and that lesion size invariably increases with power: the application of higher power leads to the direct heating of a larger zone by resistive heating and, to a lesser extent, by slow, conductive heating. This makes the lesion prone to be irreversible (24). For thin tissue, such as the LA, a higher power can consistently lead to broader and irreversible transmural lesions, improving lesion-to-lesion uniformity and durability in contiguous lesion cooking, avoiding gaps in PV encirclement. This is very important for certain sites for which catheter manipulation is difficult, for example, the roof and anterior ridge of the left PV, where consistent contact force over the PV ostia is difficult, and fast heating and a larger core of resistant heating can potentially help achieve better lesion quality.

An important note must be made with respect to the safety aspect of HP ablation; the safety margin was relatively low because of the fast and irreversible lesion formation. However, we decreased the targeted AI as presented in the CLOSE protocol for safety concerns. One patient with PV stenosis was observed in the HP-AI group because additional ablation was performed inside the PV to eliminate the residual PV potential. Caution is needed when using the HP-AI technique.

Another interesting finding in this study is that more patients with CTI ablation have recurrence. The effect of additional prophylaxis CTI ablation on the prognosis of patients with atrial fibrillation ablation is still controversial. Lim et al. (25) found that additional CTI ablation after PVI can improve the prognosis of patients with atrial fibrillation ablation. However, study by Pontoppidan et al. (26) and Kim et al. (27) did not reveal additional benefit with prophylaxis CTI ablation. Even in the study of Lee et al. (28), additional CTI ablation can be problematic. However, a history of AFL or the occurrence of AFL during ablation were predictors of symptomatic AFL during follow-up in AF patients undergoing PVI without CTI block ablation (29). Therefore, current guideline (30) recommend CTI ablation in addition to AF ablation in patients with previous documented or induced typical type AFL. And we followed the above recommendation in this study. Similar to the result of study by Moreira et al. (31), even though, additional CTI ablation was performed for AF patients with AFL, more recurrence observed in these patients. The reasons may be as follows: Even if CTI is blocked acutelly in operation, the recovery of CTI conduction after operation is not uncommon, which may lead to the recurrence of atrial arrhythmia in some patients. AFL can be a sign of advanced remodeling of both atria in patients with AF who also have a history of sustained common-type AFL (31).

### Clinical implications

In a milder AI setting for AF ablation, which is widely applied in Asian countries, HP-AI results not only in a shorter ablation time but also in better clinical AF/AFL-free survival.

### Limitations

The study has several limitations: (1) This study was conducted in a single center, with a limited number of patients; however, the sample size is comparable to that of other studies conducting AI-guided ablation (4, 6, 12, 15); (2) The study was conducted prospectively; however, the characteristics between groups were different in some aspects. Patients in the HP-AI ablation group have more extra-PV triggers and larger left atrium. These are related. Extra-PV triggers were commonly found in AF patients with significant remodeling atria which tend to be larger as reported by Kim et al. (32). However, presence of extra-PV trigger (even ablated in the index procedure) and larger LA showed a worse outcome (33). In contrast, we saw more patients maintaining sinus rhythm in the HP ablation group.; (3) Endoscopic evaluation or thermal monitoring of the esophagus was not performed because a previous study showed that important esophageal lesions did not occur beneath an AI of 400 on the posterior wall (34); and (4) The present study focused on procedure characteristics and short-term outcomes. Continued follow-up is needed to evaluate long-term efficacy.

## Conclusion

In a milder AI setting, HP-AI ablation might result in significantly higher arrhythmia-free survival than low power-AI ablation with a similar safety profile.

## Data availability statement

The raw data supporting the conclusions of this article will be made available by the authors, without undue reservation.

## Ethics statement

The studies involving human participants were reviewed and approved by the Ethics Committee of Beijing Chaoyang Hospital. The patients/participants provided their written informed consent to participate in this study.

## Author contributions

ZL: manuscript drafting and study conceptualization. L-fL: data contribution and manuscript drafting. X-qL and M-lC: study conceptualization. JL and Y-xW: follow-up and manuscript drafting. YL: data analysis and proofreading. X-pL and X-cY: study conceptualization and proofreading. All authors contributed to the article and approved the submitted version.

## Conflict of interest

The authors declare that the research was conducted in the absence of any commercial or financial relationships that could be construed as a potential conflict of interest.

## Publisher's note

All claims expressed in this article are solely those of the authors and do not necessarily represent those of their affiliated organizations, or those of the publisher, the editors and the reviewers. Any product that may be evaluated in this article, or claim that may be made by its manufacturer, is not guaranteed or endorsed by the publisher.
